# Literary Notes

**Published:** 1900-02

**Authors:** 


### LITERARY NOTES.

THE *North Carolina Medical Journal*, now published at Charlotte, has been made a monthly, after being published semi-monthly for several years. This sterling magazine has, we think, made a wise change and we wish it continued success in its new form.

THE *Archives of Pediatrics* changed editors at the beginning of the present year. Dr. Floyd M. Crandall, who has ably conducted the magazine for some years, resigned, and Dr. Walter Lester Carr has succeeded him. The price of the *Archives* is reduced to $2.00 a year.

THE *Maryland Medical Journal*, heretofore published as a weekly will hereafter appear monthly. We think the change a commendable one. The first monthly number, January, 1900, presents an excellent appearance, is well dressed and contains good material. The traditions of this staunch publication are well maintained in its new form.

THE *Providence Medical Journal* made its first appearance in January, 1900. It is edited and published by the Providence Medical Association and will appear once in each quarter year. It is announced that only original matter will be printed in it, that is to say it will contain no reprinted or abstracted material. It announces editorially that "it has nothing to sell and no favors to grant." The first number containing thirty-six pages makes an excellent appearance, and we wish the journal unbounded success on the lines it has marked out for itself.

THE American Medical Association offers an annual prize consisting of a gold medal, for meritorious scientific work. The commercial value of the medal is $100, and is awarded by a committee of which Dr. G. M. Gould, of Philadelphia, is chairman. Competing essays must be in the hands of the committee on or before March 1, 1900.

THE *Iris* for 1900 is well under way. This publication by the undergraduates of the University of Buffalo is also a work of great interest to the alumni of the University. The committee in charge of editing and publishing it are desirous of securing the coöperation of the alumni. The price of this book is $2.00, in advance, and will be ready for delivery March 1, 1900. Subscriptions should be sent to Beatrice A. Todd, class of 1900, Medical Department, University of Buffalo.

---

W. B. SAUNDERS, medical publisher, 925 Walnut Street, Philadelphia, announces that Saunders's American Year Book of Medicine and Surgery for 1900 will be issued in two volumes, instead of one as heretofore. One volume will be devoted to medicine and one to surgery, each will be complete in itself, and the work will be sold separately or in sets. Prices per volume, cloth, $3.00 net; half morrocco, $3.75 net.

---

MESSRS. P. BLAKISTON'S SON & CO., of 1012 Walnut Street, Philadelphia, announce the publication of A treatise on human anatomy in its application to the practice of medicine and surgery, by John B. Deaver, M. D., surgeon-in-chief to the German Hospital, Philadelphia, formerly assistant professor of applied anatomy in the University of Pennsylvania. The work will consist of three royal octavo volumes, 450 full page plates. Handsome cloth, $21.00; full sheep, $24.00; half green morocco marbled edges, $24.00; half russia, gilt, marbled edges, $27.00 net. Volume I. now ready; 152 full page plates. Regions considered: Upper extremity, back of neck, shoulder and trunk, cranium, scalp, face.

This volume has been published and the first printing for the most part sold. Both in its text and illustrations it has equaled every expectation,. The purpose to publish a work which makes the study of applied anatomy easy, interesting and of great usefulness to the practising physician and surgeon has been unquestionably attained.

The second volume is now on press and will be published in February, 1900. The regions described are : Neck, mouth, pharynx, larynx, nose, orbit, eyeball, organ of hearing, brain, male perineum, female perineum.

The third volume is in preparation and nearly ready for press. The regions described are : Abdominal wall, abdominal cavity, pelvic cavity, chest, lower extremity, and will include about 150 full page plates.

MESSRS. LEA BROTHERS & Co., of Philadelphia, announce that the volumes of Series of Pocket-text Books will hereafter be bound in red cloth, heavy beveled edge boards, and also in flexible red leather with round corners and with margins trimmed to facilitate carrying in the pocket. The leather bound books will cost 50 cents more than the cloth bound.

---

MESSRS. E. B. TREAT & Co., medical publishers, New York, announce that the 1900 edition of the International Medical Annual, will soon be issued. They also say that never before has there been connected with the issuance of the Annual, as editors and contributors, such an array of distinguished authorities in the various departments of medicine and surgery.

They further confidently assure their patrons and friends that the 1900 Medical Annual will be better by far than its predecessors; and that it will come as in previous years (to use the words of the *New York Medical Record*) "as an annual treat, covering the advances in the several departments of medical science."

